# Distinguishing tautomerism in the crystal structure of (*Z*)-*N*-(5-ethyl-2,3-di­hydro-1,3,4-thia­diazol-2-yl­idene)-4-methyl­benzene­sulfonamide using DFT-D calculations and ^13^C solid-state NMR

**DOI:** 10.1107/S2053229614015356

**Published:** 2014-07-19

**Authors:** Xiaozhou Li, Andrew D. Bond, Kristoffer E. Johansson, Jacco Van de Streek

**Affiliations:** a Department of Pharmacy, University of Copenhagen, Universitetsparken 2, Copenhagen DK-2100, Denmark

**Keywords:** crystal structure, powder diffraction, NMR analysis, amine–imine tautomerism, dispersion-corrected DFT

## Abstract

The crystal structure of the title compound, C_11_H_13_N_3_O_2_S_2_, has been determined previously on the basis of refinement against laboratory powder X-ray diffraction (PXRD) data, supported by comparison of measured and calculated ^13^C solid-state NMR spectra [Hangan *et al.* (2010 ▶). *Acta Cryst.* B**66**, 615–621]. The mol­ecule is tautomeric, and was reported as an amine tautomer [systematic name: *N*-(5-ethyl-1,3,4-thia­diazol-2-yl)-*p*-toluene­sulfonamide], rather than the correct imine tautomer. The protonation site on the mol­ecule’s 1,3,4-thia­diazole ring is indicated by the inter­molecular contacts in the crystal structure: N—H⋯O hydrogen bonds are established at the correct site, while the alternative protonation site does not establish any notable inter­molecular inter­actions. The two tautomers provide essentially identical Rietveld fits to laboratory PXRD data, and therefore they cannot be directly distinguished in this way. However, the correct tautomer can be distinguished from the incorrect one by previously reported qu­anti­tative criteria based on the extent of structural distortion on optimization of the crystal structure using dispersion-corrected density functional theory (DFT-D) calculations. Calculation of the ^13^C SS-NMR spectrum based on the correct imine tautomer also provides considerably better agreement with the measured ^13^C SS-NMR spectrum.

## Abstract 2

The crystal structure of (*Z*)-*N*-(5-ethyl-2,3-di­hydro-1,3,4-thia­diazol-2-yl­idene)-4-methyl­benzene­sulfonamide contains an imine tautomer, rather than the previously reported amine tautomer. The tautomers can be distinguished using dispersion-corrected density functional theory calculations and by comparison of calculated and measured ^13^C solid-state NMR spectra.

## Introduction   

Determination of mol­ecular crystal structures from powder X-ray diffraction (PXRD) data is now relatively common in the chemical literature (see, for example, Sanphui *et al.*, 2014 ▶; Madusanka *et al.*, 2014 ▶; Braun *et al.*, 2013 ▶; Smart *et al.*, 2013 ▶). On account of the compression of the diffraction data into one dimension in the powder pattern, it is frequently necessary, and in any case always of value, to supplement refinement against PXRD data with independent information that can establish the correctness of the structure or provide a more reliable indication of features that cannot be distinguished from the PXRD data alone. Energy minimization using quantum-chemical calculations provides one option, which can be especially useful for the determination of accurate positions for H atoms (Deringer *et al.*, 2012 ▶). Energy minimization of a correct experimental crystal structure should lead to relatively smaller distortions compared to minimization of an incorrect structure, thereby providing possibilities for qu­anti­tative structure validation (Van de Streek & Neumann, 2010 ▶). Another possibility is *ab initio* calculation of solid-state nuclear magnetic resonance (SS-NMR) spectra for comparison to experimental spectra. Progress in this area is developing into the subfield of ‘NMR crystallography’ (Harris *et al.*, 2009 ▶).
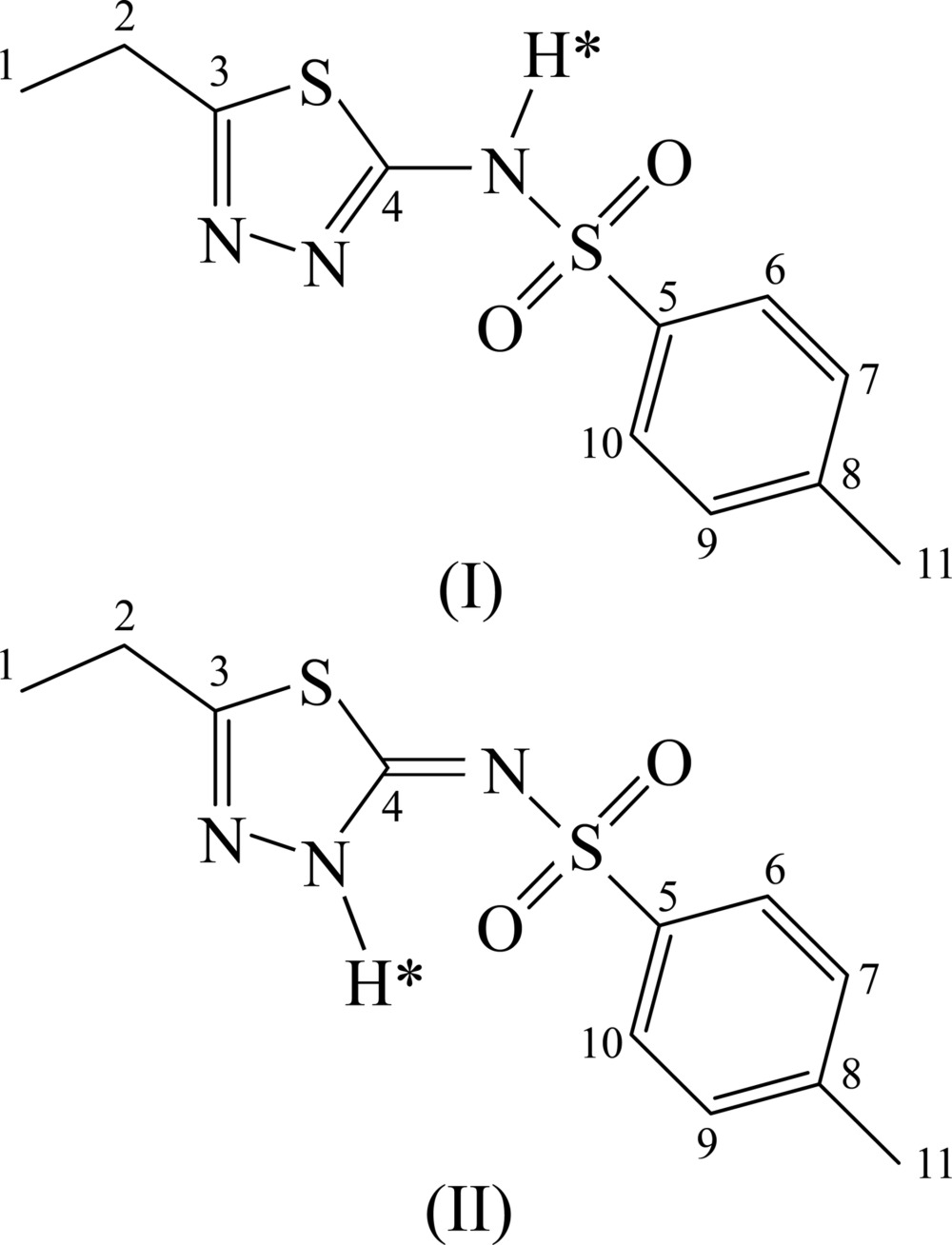


**Figure 1:**
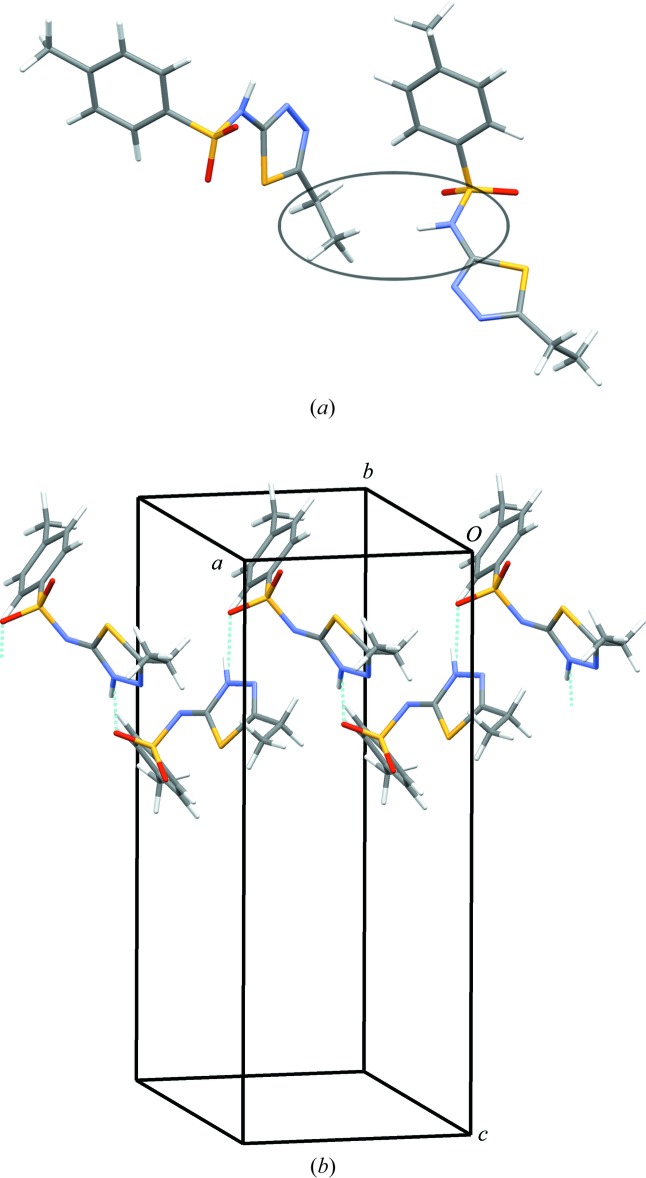
Inter­molecular inter­actions for (*a*) amine tautomer (I), the N—H group points directly towards a neighbouring methyl group, and (*b*) imine tautomer (II), where inter­molecular N—H⋯O hydrogen bonds generate ribbons along the *a* axis.

The crystal structure of the title compound has been reported by Hangan and co-workers [Hangan *et al.*, 2010 ▶; Cambridge Structural Database (CSD; Allen, 2002 ▶) refcode UKIRAI] on the basis of refinement against laboratory PXRD data, supplemented by comparison of measured and calculated ^13^C SS-NMR spectra. The compound is tautomeric, with alternative H-atom positions on the N atom external to the 1,3,4-thia­diazole ring [referred to as the amine tautomer, (I)] or on one N atom of the 1,3,4-thia­diazole ring [referred to as the imine tautomer, (II)]. Hangan *et al.* reported the crystal structure and accompanying ^13^C SS-NMR calculations on the basis of the amine tautomer (I). Looking at the structure, there is an indication that this might not be correct: the postulated amine N—H group points directly towards a neighbouring methyl group (Fig. 1 ▶ *a*), while the N atom at the alternative protonation site on the 1,3,4-thia­diazole ring forms a contact of 2.78 Å to an O atom in a neighbouring mol­ecule. Thus, it seems more likely that the imine tautomer (II) is present in the crystal structure, with the N—H group forming inter­molecular N—H⋯O hydrogen bonds that generate ribbons along the *a* axis (Fig. 1 ▶ *b*).

It seems unlikely that the H-atom positions could be determined explicitly by refinement against the laboratory PXRD data (*vide infra*), so it is inter­esting to examine the extent to which the tautomers can be distinguished by complementary methods, and especially the published ^13^C SS-NMR data. The discrepancy between observed and calculated ^13^C SS-NMR chemical shifts obtained by Hangan *et al.* for the amine tautomer (I) [mean absolute deviation (MAD) = 7.70 p.p.m. and root-mean-square deviation (RMSD) = 8.95 p.p.m.] is quite large compared to others in the literature (see, for example, Kuttatheyil *et al.*, 2013 ▶; Dudenko *et al.*, 2013 ▶; Filip *et al.*, 2013 ▶) and the agreement could potentially be improved by considering the imine tautomer (II). In this paper, we reconsider the published experimental data (PXRD and ^13^C SS-NMR), together with some additional geometry optimizations and solid-state NMR calculations based on dispersion-corrected density functional theory (DFT-D) calculations. The aim is to establish the extent to which the tautomers might be distinguishable in the solid state.^1^ It is shown that the imine tautomer (II) is present in the crystal structure, and that tautomers (I) and (II) can be qu­anti­tatively distinguished by the results of DFT-D geometry optimizations and the published ^13^C SS-NMR spectra.

## Experimental   

### Structure refinement   

Crystal data, data collection and structure refinement details are summarized in Table 1 ▶. Structure refinements were carried out with *TOPAS Academic* (Coelho, 2012 ▶), using the PXRD data of Hangan *et al.* (2010 ▶). The published crystal structure was used as a starting point and H atoms were added in calculated positions. Two models were made, corresponding to tautomers (I) and (II), then both were subjected to preliminary DFT-D energy minimization with all atoms and unit-cell parameters free to vary within the constraints of the space group *Pbca*. This first step provides optimized models from which to extract restraints on the mol­ecular geometry (Naelapää *et al.*, 2012 ▶). The model structures for (I) and (II) were then subjected to Rietveld refinement against the published data, with restraints on the intra­molecular bond distances and angles taken from the DFT-D calculations. The applied restraints are slightly different for each refinement, and the refined models therefore differ slightly where the bond lengths are influenced by the tautomeric form. Since the positions of the H atoms refined against the laboratory PXRD data are uncertain, a final *CASTEP* optimization (energy cut-off = 520 eV) was applied, with only the H atoms allowed to move. The final experimental structures for (I) and (II) have the unit cell and non-H-atom positions obtained from the Rietveld refinement, with the *CASTEP*-optimized positions for the H atoms.

### DFT-D optimizations and calculation of ^13^C SS-NMR spectra   

**Figure 2:**
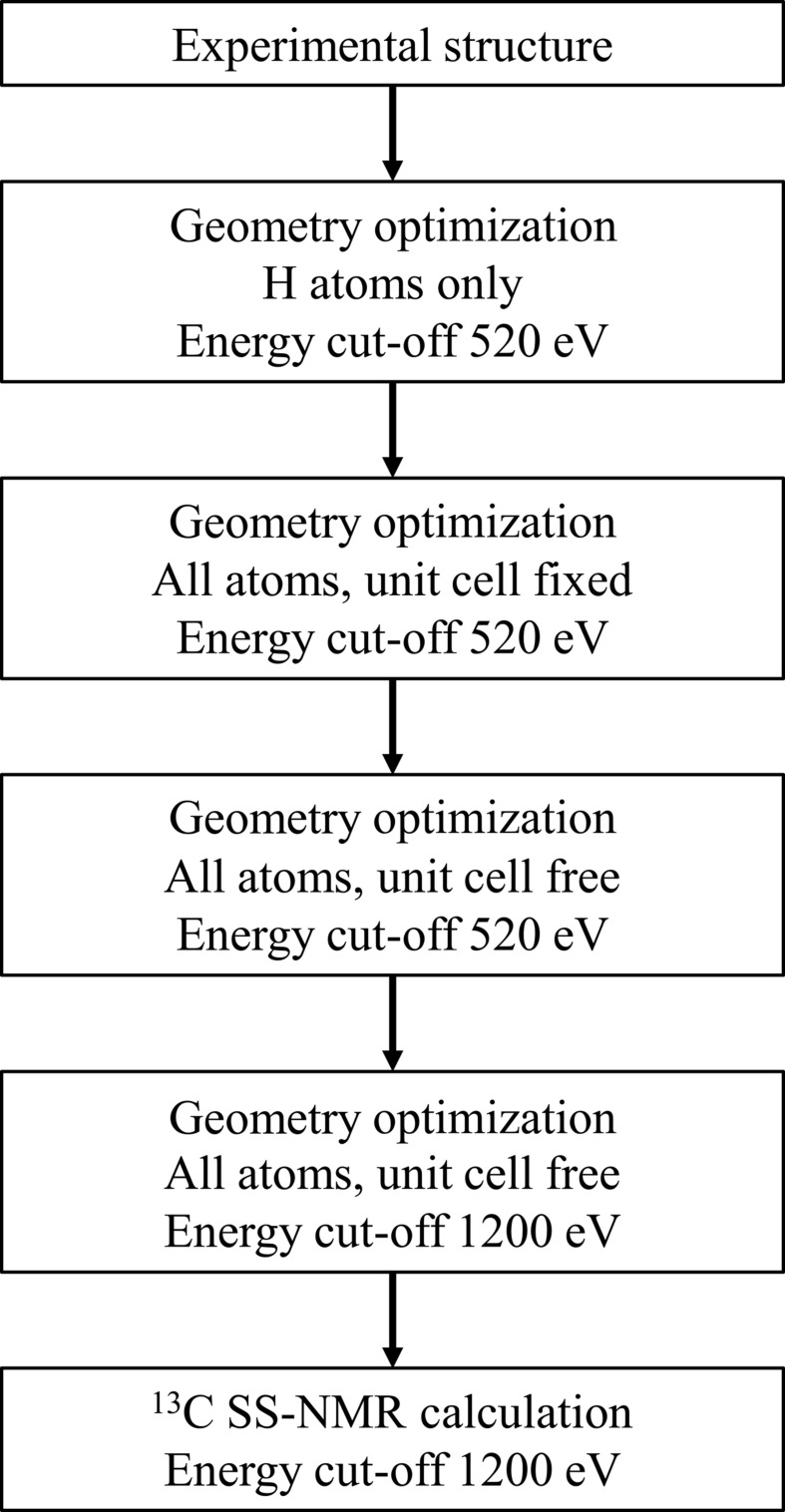
Protocol for the structure optimizations and calculations of the ^13^C SS-NMR spectra.

Geometry optimizations and solid-state NMR calculations were carried out using *CASTEP* (Academic Release 6.1; Clark *et al.*, 2005 ▶), *via* the inter­face within *Materials Studio* (Accelrys, 2011 ▶). The Perdew, Burke and Ernzerhof (PBE) exchange-correlation functional (Perdew *et al.*, 1996 ▶) was applied, with the Grimme-06 semi-empirical dispersion correction (Grimme, 2006 ▶). Integrals taken over the Brillouin zone were carried out on a Monkhorst–Pack grid (Monkhorst & Pack, 1976 ▶) with a maximum sample spacing of 0.05 Å^−1^. For both (I) and (II), the experimental structures were optimized and ^13^C SS-NMR spectra were calculated by following the flowchart shown in Fig. 2 ▶. In general, an optimization of the crystal structure is divided into three sequential steps with: (i) only H atoms allowed to move; (ii) all atoms allowed to move with unit-cell parameters fixed; (iii) all atoms allowed to move with unit-cell parameters free. The optimizations were carried out with an energy cut-off of 520 eV to permit comparison with the results of a published validation study (Van de Streek & Neumann, 2010 ▶). The optimized structure at 520 eV with the unit cell free was further optimized with an energy cut-off of 1200 eV and then used for ^13^C SS-NMR calculations at 1200 eV, with ultrasoft pseudopotentials generated on-the-fly (Yates *et al.*, 2007 ▶). Optimization with the higher-quality basis set is more time-consuming, but provides a more accurate calculated ^13^C SS-NMR spectrum.

In order to isolate the influence of the H-atom positions on the calculated ^13^C SS-NMR spectra, an ‘average structure’ was prepared from the experimental structures of (I) and (II), by averaging each corresponding unit-cell parameter and the atomic coordinates of the non-H atoms. The unit cell and non-H atoms of this model are not biased towards either tautomer. The H atoms were then placed so as to form either tautomer (I) or (II) and their positions were optimized with an energy cut-off of 1200 eV, with the unit cell and non-H-atom positions fixed. These two structures are referred to as ‘average (I)’ and ‘average (II)’, respectively. ^13^C solid-state NMR calculations were made for the two structures with an energy cut-off of 1200 eV.

## Results and discussion   

### Structure refinement against PXRD data   

**Figure 3:**
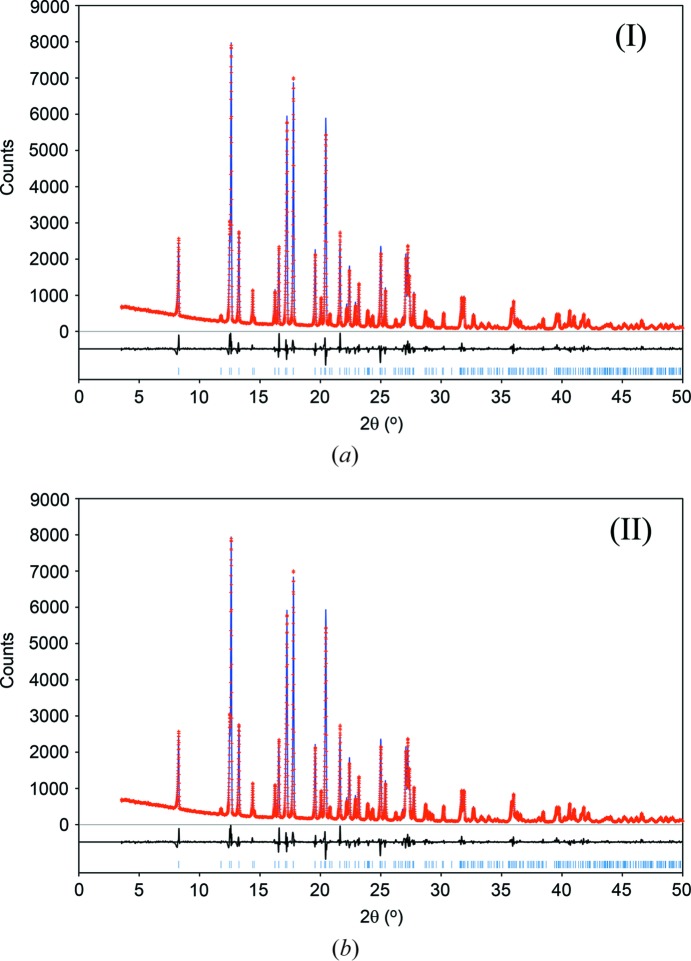
Rietveld plots for the refined experimental structures of (I) and (II). The PXRD data are taken from Hangan *et al.* (2010 ▶). Key: red crosses = measured data, blue line = calculated pattern and black line = difference curve.

Structure refinement against the PXRD data using either tautomer (I) or (II) produced essentially identical results (Table 1 ▶ and Fig. 3 ▶). The obtained figures-of-merit are moderately improved compared to the refinement of Hangan *et al.* (2010 ▶), principally due to improved anisotropic peak-shape modelling, and inclusion of a preferred-orientation correction [March, 1932 ▶; direction [100], refined parameter *ca* 1.08 for both (I) and (II)]. Nonetheless, the features in the difference curves reveal remaining problems with the peak shape, which possibly play some role in obscuring any differences that might have been evident between the two models. Using the present PXRD data, we conclude that it is not possible to distinguish directly the two tautomers.

### Structure validation by DFT-D optimization   

**Figure 4:**
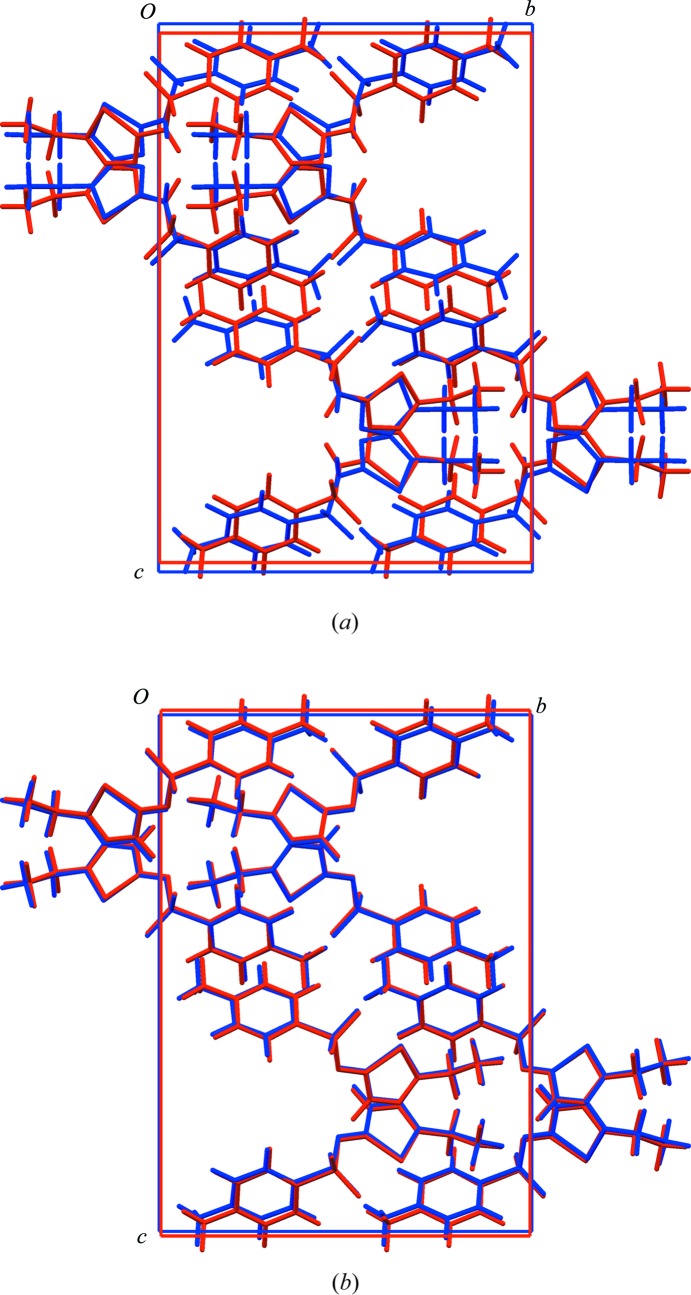
Overlay of experimental structure (red) and optimized structure (blue) at 520 eV with the unit-cell dimensions free. The larger distortion for (I) can be seen clearly.

The results of DFT-D energy minimizations at 520 eV for the experimental structures of (I) and (II) are shown in Table 2 ▶. The input and optimized structures in CIF format are provided as *Supporting information*. On minimization, structure (I) undergoes significantly larger distortions compared to structure (II), both when the unit cell is fixed to the experimental one and when it is free to be optimized. The difference between (I) and (II) is visually evident in overlays of the experimental and optimized structures (Fig. 4 ▶). According to a previous validation study (Van de Streek & Neumann, 2010 ▶), an RMS Cartesian displacement for the non-H atoms greater than 0.30 Å when the unit-cell parameters are allowed to vary indicates either that the structure is incorrect, or that it undergoes some significant temperature-dependent variation. Values below 0.25 Å indicate that the structure is likely to be correct. These established geometrical criteria indicate that structure (II) is acceptable and identify structure (I) as suspicious (Table 2 ▶).

### ^13^C Solid-state NMR   

The results of our ^13^C SS-NMR calculations are listed in Table 3 ▶. The resonance assignment is based on that of Hangan *et al.*, which was made by comparison with the solution ^13^C spectrum. For the topologically-equivalent atom pairs C6/C10 and C7/C9, single resonances in the solution spectrum are split into two pairs in the solid state, and it is not possible to state *a priori* which resonance corresponds to which atom within each pair. In these cases, the assignment is made so as to provide the best fit with the experimental spectrum. For (I), and for the two average structures, the assignment corresponds to that of Hangan *et al.* For (II), the agreement with the experimental spectrum is improved by exchanging the assignment of C6 and C10 (see Table 3 ▶). Overall, the calculated chemical shifts of (II) give better agreement with the experimental chemical shifts than do the calculated shifts of (I). The RMSD value of 1.9 p.p.m. for (II) is identical to a mean value obtained for similar test calculations on 15 organic compounds (Salager *et al.*, 2010 ▶), so it can be viewed as being compatible with established expectations. By contrast, the RMSD value of 4.0 p.p.m. for (I) is significantly larger than expectation. The largest single deviation for (II) is −3.4 p.p.m. for atom C11, compared to 10.7 p.p.m. for atom C3 in (I). The latter error is clearly substantial, and indicative of the incorrect tautomeric assignment. Thus, comparison of the published ^13^C SS-NMR data with our new calculations clearly distinguishes the two tautomers.

For the models made by averaging the non-H-atom positions in the two experimental structures, the MAD and RMSD values for (I) and (II) are quite similar, and both have some atoms with relatively large deviations (> 4 p.p.m.) compared to the measured chemical shifts (Table 3 ▶). This demonstrates that the calculated ^13^C chemical shifts are very sensitive to the positions of all atoms, rather than just the H-atom positions in this tautomeric case. The averaged model provides a better fit compared to the fully optimized structure for the incorrect tautomer (I) but a worse fit for the correct tautomer (II). This is related to the ‘tautomer-dependent’ restraints that were applied in the Rietveld refinements, derived from preliminary DFT-D optimizations (see *Experimental*, §2.2). For (I), the preliminary DFT-D optimization was influenced by the incorrect choice of H atom positions, and therefore provided relatively inaccurate positions for the non-H atoms. These positions are carried over into the experimental structures through the applied restraints. For (II), the preliminary DFT-D optimization provided more accurate positions for the non-H atoms because of the correct choice for the H atoms. Averaging of the two experimental structures moves the relatively poor heavy-atom positions in the incorrect structure (I) towards the better heavy-atom positions in the correct structure (II), thereby giving progressively improved fits to the ^13^C SS-NMR data in the sequence (I) → average (I) → average (II) → (II).

## Conclusions   

The crystal structure of the title compound contains the imine tautomer (II) rather than the previously reported amine tautomer (I). The tautomers cannot be directly distinguished from the laboratory PXRD data (although the inter­molecular contacts in the crystal structure are strongly indicative of the imine tautomer); so this is a case in which independent qu­anti­tative information becomes useful. The aim of this paper was to reconsider the published information, rather than to collect any new experimental data (*e.g.* ^1^H SS-NMR data), with the addition of some computational work. The incorrect tautomer is highlighted by DFT-D optimization, on the basis of criteria presented in an earlier validation study (Van de Streek & Neumann, 2010 ▶). This computational procedure is relatively accessible, and we suggest that it should always be used to support mol­ecular crystal structures determined from PXRD data. Comparison of the calculated and published ^13^C solid-state NMR spectra also provides a clear qu­anti­tative distinction between the two tautomers.

## Supplementary Material

Crystal structure: contains datablock(s) global, I. DOI: 10.1107/S2053229614015356/fa3347sup1.cif

Rietveld powder data: contains datablock(s) I. DOI: 10.1107/S2053229614015356/fa3347Isup2.rtv

Input and optimized structure for (I). DOI: 10.1107/S2053229614015356/fa3347Isup3.txt

Input and optimized structure for (II). DOI: 10.1107/S2053229614015356/fa3347Isup4.txt

CML file for (I). DOI: 10.1107/S2053229614015356/fa3347Isup5.cml

CCDC reference: 1011334

## Figures and Tables

**Table 1:** Experimental details The experimental data were taken from Hangan *et al.* (2010 ▶).

	(I)	(II)
Crystal data		
Chemical formula	C_11_H_13_N_3_O_2_S_2_	C_11_H_13_N_3_O_2_S_2_
*M*_r_	283.36	283.36
Crystal system, space group	Orthorhombic, *P* *b* *c* *a*	Orthorhombic, *P* *b* *c* *a*
Temperature (K)	298	298
*a*, *b*, *c* (Å)	8.53925 (14), 15.0207 (3), 21.3958 (3)	8.53937 (13), 15.0206 (2), 21.3960 (3)
*V* (Å^3^)	2744.33 (8)	2744.39 (7)
*Z*	8	8
Radiation type	Cu *K*α_1_, λ = 1.54056 Å	Cu *K*α_1_, λ = 1.54056 Å
Specimen shape, size (mm)	Flat sheet, 25 × 1	Flat sheet, 25 × 1
	
Data collection		
Diffractometer	Bruker D8 Advance diffractometer	Bruker D8 Advance diffractometer
Specimen mounting	Bruker sample cup	Bruker sample cup
Data collection mode	Reflection	Reflection
Scan method	Continuous	Continuous
2θ values (°)	2θ_min_ = 3.54, 2θ_max_ = 50.03, 2θ_step_ = 0.005	2θ_min_ = 3.54, 2θ_max_ = 50.03, 2θ_step_ = 0.005
	
Refinement		
*R* factors and goodness of fit	*R*_p_ = 0.058, *R*_wp_ = 0.081, *R*_exp_ = 0.053, *R*(*F*) = 0.034, χ^2^ = 1.53	*R*_p_ = 0.058, *R*_wp_ = 0.081, *R*_exp_ = 0.053, *R*(*F*) = 0.033, χ^2^ = 1.53
No. of data points	9298	9298
No. of parameters	127	127
No. of restraints	88	88
H-atom treatment	H-atom parameters not refined	H-atom parameters not refined

Computer programs: *TOPAS Academic* (Coelho, 2012 ▶) and *Mercury* (Macrae *et al.*, 2008 ▶).

**Table 2:** Root-mean-square Cartesian displacements (Å) for the DFT-D optimizations of (I) and (II), compared to the experimental structures

	Energy cut-off (eV)	Optimization protocol	All-atom RMS Cartesian displacement	Non-H-atom RMS Cartesian displacement
(I)	520	Unit cell fixed	0.4309	0.3487
(I)	520	Unit cell free	0.4356	0.3561
(I)	1200	Unit cell free	0.6972	0.5749
				
(II)	520	Unit cell fixed	0.1494	0.1283
(II)	520	Unit cell free	0.1569	0.1329
(II)	1200	Unit cell free	0.1626	0.1282

**Table 3:** Experimental and calculated ^13^C S*S*-NMR chemical shifts (p.p.m.) Deviations compared to the experimental values are indicated in parentheses. All calculations are based on optimized structures at 1200 eV (as described in the text) and are carried out at 1200 eV.

	Experimental^*a*^	(I)		(II)		Average (I)		Average (II)	
C1	14.0	11.8	(−2.2)	11.8	(−2.2)	13.2	(−0.8)	10.9	(−3.1)
C2	23.5	19.4	(−4.1)	20.9	(−2.6)	20.6	(−2.9)	22.1	(−1.4)
C3	165.7	176.4	(10.7)	168.2	(2.5)	165.4	(−0.3)	161.4	(−4.3)
C4	161.9	165.4	(3.5)	163.1	(1.2)	159.3	(−2.6)	162.6	(0.7)
C5	138.9	138.9	(0.0)	140.3	(1.4)	140.4	(1.5)	140.6	(1.7)
C6	127.6	125.5	(−2.1)	128.1	(–0.2)*^*b*^*	126.2	(−1.4)	128.4	(0.8)
C7	130.5	128.7	(−1.8)	131.6	(1.1)	132.5	(2.0)	132.0	(1.5)
C8	145.0	147.4	(2.4)	147.1	(2.1)	148.9	(3.9)	148.2	(3.2)
C9	132.0	131.5	(−0.5)	133.3	(1.3)	136.6	(4.6)	135.0	(3.0)
C10	128.3	125.9	(−2.4)	126.7	(−0.9)^*b*^	126.9	(−1.4)	128.5	(0.2)
C11	21.3	17.8	(−3.5)	17.9	(−3.4)	18.9	(−2.4)	19.3	(−2.0)
MAD			3.0		1.7		2.2		2.0
RMSD			4.0		1.9		2.5		2.3

Notes: (*a*) experimental values and resonance assignments from Hangan *et al.* (2010 ▶); (*b*) the assignments of the topologically equivalent atoms C6 and C10 are exchanged: C6 is matched to the experimental value of C10, and *vice *versa** (see main text).

## Footnotes

1 The paper of Hangan *et al.* does not contain a ^1^H SS-NMR spectrum. A ^1^H NMR spectrum in *d*_6_-DMSO solution is mentioned, but caution should be applied when extrapolating solution data to the solid state, because protonation states and hydrogen bonding may be different (*e.g.* barbituric acid; Schmidt *et al.*, 2011 ▶).
